# University students’ exercise intention in the context of technology assistance: testing an integrated model of SDT and TPB

**DOI:** 10.3389/fpsyg.2025.1700121

**Published:** 2025-11-14

**Authors:** Jingyi Wang, Syed Kamaruzaman Bin Syed Ali, Wenting Bao

**Affiliations:** 1 Department of Educational Foundations and Humanities, Universiti Malaya Faculty of Education, Federal Territory of Kuala Lumpur, Malaysia; 2 School of Humanities and International Education Exchange, Anhui University of Chinese Medicine, Hefei, China

**Keywords:** perceived behavior control, campus running check-in app, novelty, variety, basic psychological need

## Abstract

**Purpose:**

This study explores the psychological mechanisms underlying university students’ intentions to engage in technology-assisted exercise, focusing on campus running applications for university physical exercise management in China.

**Method:**

Guided by an integrated Self-Determination Theory (SDT) and Theory of Planned Behavior (TPB) framework, data were collected from 477 undergraduates. Five psychological needs—autonomy, competence, relatedness, novelty, and variety—were examined as predictors of exercise intention, with attitude, subjective norms, and perceived behavioral control as mediators. Partial Least Squares Structural Equation Modeling (PLS-SEM) was used for analysis.

**Results:**

The findings showed that basic psychological needs significantly predicted exercise intention. Perceived behavioral control was the strongest mediator, whereas subjective norms had no significant influence.

**Discussion/conclusion:**

This study highlights the roles of novelty, variety, and perceived control in sustaining motivation, refining SDT–TPB integration, and informing the design of digital health interventions for students.

## 1 Introduction

University is a pivotal stage in the shift from school-based physical education to lifelong activity. Well-designed systematic physical education (PE) can sustain and improve aerobic endurance, bone development, and cardiorespiratory fitness. It also buffers academic stress and strengthens psychological resilience and social adaptation. Despite these benefits, many universities still provide limited PE offerings. As a result, almost 60% of students fail to meet World Health Organization activity guidelines (Bailey et al., 2023). This inactivity is closely linked to increasing rates of obesity, depression, and other chronic conditions in young adults (Watson et al., 2022).

In response, technology-assisted exercise solutions have emerged and rapidly gained popularity. While some students occasionally use online platforms such as Youku or Bilibili to follow guided workout videos, these alternatives are typically informal and self-initiated. In contrast, the campus running application remains the most widely used digital exercise system among Chinese university students because it is institutionally integrated, linked to course assessments, and officially endorsed by universities. Recent surveys show the wide adoption of digital tools, including wearable trackers (Ráthonyi et al., 2019), immersive Virtual Reality (VR) exercise platforms (Liu et al., 2024), and mobile physical activity applications (Wang et al., 2019). However, access does not guarantee persistence. A review of about 526,000 adult users found that a median of 70% abandon health-and-fitness apps within the first 100 days, leaving fewer than one-third active after 3 months (Kidman et al., 2024). Such findings suggest that technological novelty alone may initially attract users but fail to ensure enduring engagement, emphasizing the need to explore deeper motivational factors. Therefore, identifying the psychological and cognitive mechanisms that support students’ exercise intentions in digital settings is a pressing research priority.

In China, sedentary behavior among university students is exacerbated by limited physical education and academic pressure. A nationwide survey of university students from six provinces found that only 4.8% met the World Health Organization’s guideline of ≥60 min physical activity per day (Deng et al., 2024). Recent national monitoring data further indicate that although nearly 80% of students have downloaded digital fitness or running applications, fewer than 35% have used them consistently for more than a month (China Youth Daily, 2023). Surveys based on the Self-Determination Theory framework show moderate levels of competence satisfaction but low autonomy and relatedness among digital exercisers, suggesting limited internalization of exercise motivation and unstable behavioral intentions.

To curb this trend, many universities have introduced campuses running check-in applications as part of the National Student Fitness Assessment System. In most institutions, participation is semi-mandatory because recorded running data directly contribute to students’ physical education grades or attendance evaluations. Universities typically establish specific policies regarding the number of weekly check-ins, minimum running distance, and compliance monitoring, which formalize the app’s role as both a fitness tool and an assessment mechanism (Wang et al., 2019). Building upon these national initiatives, digital health solutions, especially campus running check-in applications launched under the Healthy China 2030 strategy, have become widespread (Liu et al., 2024). While some students occasionally use online platforms such as Youku or Bilibili to follow guided workout videos, these alternatives are typically informal and self-initiated. In contrast, the campus running application remains the most widely used digital exercise system among Chinese university students because it is institutionally integrated, linked to course assessments, and officially endorsed by universities (Wan et al., 2024).

Existing theoretical frameworks offer only partial explanations. The Theory of Planned Behavior (TPB) effectively captures cognitive predictors such as attitude, subjective norms, and perceived behavioral control, but it tends to overlook the intrinsic motivational processes that energize and sustain behavior (Ajzen, 1991). Conversely, Self-Determination Theory (SDT) elucidates how satisfaction of autonomy, competence, and relatedness fosters self-driven engagement, but it lacks specificity in predicting intention formation within structured digital contexts (Deci and Ryan, 2000). Therefore, integrating these two perspectives allows for a more comprehensive understanding of both motivational quality and intentional control in students’ digital exercise behavior.

Furthermore, recent extensions of SDT have identified two additional psychological needs, novelty and variety, as essential for maintaining interest and preventing motivational fatigue in repetitive physical activities (González-Cutre et al., 2016; Sylvester et al., 2014). Novelty reflects the desire to experience new and stimulating activities, whereas variety denotes a preference for diverse experiences within familiar settings. In digital exercise environments, these needs become particularly salient because features such as gamified challenges, progress badges, and rotating workout modes can continually refresh user interests and mitigate monotony (Caso et al., 2023). Thus, examining novelty and variety extends the SDT framework to better capture sustained motivation in technology-mediated contexts.

Similarly, the interactive affordances of exercise technologies directly correspond to the SDT dimensions. Gamification and real-time feedback enhance competence by providing visible progress and mastery cues; personalized goal-setting and adaptive training modules support autonomy by allowing users to choose intensity and pace; and social leaderboards and peer communities nurture relatedness by enabling connection and mutual encouragement (Deci and Ryan, 2000). These psychological mechanisms clarify why certain technological designs succeed in sustaining engagement, while others fail.

Therefore, this study bridges the two frameworks by modeling SDT needs as distal antecedents that feed into TPB’s proximal determinants. It also incorporates two additional needs, novelty and variety, as identified by González-Cutre et al. (2016) and Sylvester et al. (2014). Specifically, this study aims to

1. Examine how satisfaction of autonomy, competence, and relatedness shapes students’ intention to use technology-assisted exercise;
2. Investigate novelty and variety as distinct motivational needs;
3. Test whether TPB variables mediate the link between need satisfaction and behavioral intention.

## 2 Theoretical background

Over the last decade, scholars have increasingly combined Self-Determination Theory (SDT) and the Theory of Planned Behavior (TPB) to explain health-related actions. SDT provides a motivational foundation, whereas TPB specifies the cognitive beliefs that precede intention. The following section reviews the key SDT variables, the key TPB variables, the evidence for their integration, and the context of the present study, which tests the model among Chinese university students who use campus running “check-in” apps.

### 2.1 Self-Determination Theory

Self-Determination Theory (SDT) posits that human behavior is driven by the satisfaction of three fundamental psychological needs: autonomy (the need to experience volition), competence (the need to feel effective), and relatedness (the need to feel connected to others) (Deci and Ryan, 2000). When these needs are fulfilled, individuals are more likely to engage in activities for intrinsic reasons, leading to enhanced motivation, persistence, and well-being.

In the domain of exercise, need satisfaction has been robustly associated with sustained physical activity and self-determined motivation (Teixeira et al., 2012; Vasconcellos et al., 2020). However, in modern digital environments, especially those involving mobile health applications, users often encounter algorithmic repetition, limited interactivity, and monotonous routines. These features may inhibit long-term engagement by failing to satisfy the dynamic and experiential nature of motivation.

To address these limitations, recent theoretical developments have expanded SDT by identifying two additional needs: novelty (desire to encounter new and stimulating experiences) and variety (need to engage in diverse activities) (González-Cutre et al., 2016; Sylvester et al., 2014). The three traditional needs of SDT explain why individuals initiate and sustain activities based on autonomy, competence, and relatedness; however, they do not fully account for the motivational diversity required in repetitive digital exercise contexts. Novelty and variety respond to this gap by emphasizing stimulation and differentiation, which are critical for maintaining engagement when activities become predictable.

Empirical evidence supports this extension. Studies on digital fitness and gamification have shown that novelty and variety significantly reinforce intrinsic motivation. Fang et al. (2022) reported that introducing varied exercise challenges in mobile fitness apps increased user engagement and weekly adherence. Similarly, Looyestyn et al. (2017) demonstrated that adaptive goal systems and diversified feedback enhance user persistence by preventing motivational fatigue. These findings align with behavioral reinforcement theory and interaction design theory, which emphasize the importance of continuous stimulation, adaptive feedback, and diversity in sustaining user participation (Hamari and Koivisto, 2015; Johnson et al., 2020). Therefore, integrating novelty and variety into the SDT framework enhances its explanatory capacity in technology-mediated exercise by capturing the psychological mechanisms that sustain long-term motivation and behavioral adherence.

### 2.2 Theory of Planned Behavior

The Theory of Planned Behavior (TPB) offers a well-established cognitive framework for predicting behavioral intentions. According to the TPB, intention is directly influenced by three proximal constructs: attitude toward the behavior (positive or negative evaluations), subjective norms (perceived social pressure from others), and perceived behavioral control (belief in one’s capacity to perform the behavior) (Ajzen, 1991). TPB has demonstrated strong predictive power in health-related behaviors, including exercise and technology-assisted health management (Ajzen and Albarracín, 2007). However, TPB focuses on the cognitive formation of intentions and does not explain the motivational sources that shape these beliefs. This limitation can be addressed by linking SDT and TPB, where psychological need satisfaction provides motivational antecedents that determine attitude, subjective norms, and perceived behavioral control.

### 2.3 Integration of SDT and TPB

Researchers have increasingly integrated SDT and TPB to provide a comprehensive understanding of both motivational depth and cognitive specificity in health-related behaviors. The seminal meta-analysis by Hagger and Chatzisarantis (2009) demonstrated that SDT’s constructs of autonomy, competence, and relatedness indirectly predict behavioral intentions through the mediating effects of TPB variables such as attitude, subjective norms, and perceived behavioral control. Similar results have been observed across domains, including injury prevention (Chan and Hagger, 2012), chronic illness management (Brooks et al., 2017), and voluntary health actions such as blood donation (Williams et al., 2019). More recently, Caso et al. (2023) expanded the framework by integrating novelty into dietary interventions, demonstrating that novelty enhances adherence intentions through improved attitudes and perceived control. Pynnönen et al. (2023) and Wan et al. (2024) further confirmed that the combined SDT–TPB model effectively predicts behavioral intentions in physical activity and public health contexts (see Table 1).

**Table 1:** Summary of studies integrating SDT and TPB (2009–2024).

Author(s) and year	Population (age/region/sample size)	Domain	SDT constructs	TPB constructs	Main findings
Hagger and Chatzisarantis (2009)	Mixed samples, global (*N* ≈ 9,500)	Health behaviors	Autonomy, competence, relatedness	Attitude, SN, PBC	SDT constructs indirectly predicted intention via TPB pathways.
Chan and Hagger (2012)	Injured athletes, UK (*N* = 214)	Injury prevention	Autonomy, competence	Attitude, PBC	Autonomous motivation enhanced rehabilitation intentions via attitude and PBC.
Brooks et al. (2017)	Chronic pain patients, Australia (*N* = 312)	Exercise behavior	Autonomy, competence, relatedness	Attitude, PBC	SDT variables predicted physical activity intention via TPB mediators.
Williams et al. (2019)	General adult population, USA (*N* = 604)	Blood donation	Autonomy, competence, relatedness	Attitude, SN, PBC	SDT and TPB constructs jointly predicted blood donation intention.
Caso et al. (2023)	Adults in RCT, Italy (*N* = 180)	Dietary adherence	Autonomy, competence, novelty	Attitude, SN, PBC	Novelty enhanced dietary intentions via improved attitudes and PBC.
Pynnönen et al. (2023)	Older adults, Finland (*N* = 427)	Physical activity	Autonomy, competence, relatedness	Attitude, SN, PBC	The integrated model effectively predicted physical activity intention.
Wan et al. (2024)	Parents and children, China (*N* = 358)	COVID-19 protective behavior	Autonomy, relatedness	Attitude, SN, PBC	Motivational support promoted protective behavior through TPB constructs.

The present study extends this integration to digital exercise behavior among Chinese university students. Campuses running check-in apps in China typically involve repetitive routes, limited customization, and minimal peer interaction. Although these apps successfully monitor exercise completion, they often lack dynamic feedback or interactive diversity, leading to user fatigue and disengagement over time. This context underscores the importance of novelty and variety as motivational drivers that can refresh user interests and sustain commitment. By embedding these constructs into the integrated SDT–TPB framework, the model captures both the cognitive mechanisms of intention formation and the motivational processes that support behavioral persistence.

In addition, cultural context plays a crucial role. In collectivist societies such as China, relatedness satisfaction extends beyond personal connections to include alignment with social expectations and group norms. According to social influence theory and cultural psychology (Cialdini and Goldstein, 2004; Triandis, 2001), individuals in collectivist contexts are more likely to internalize the expectations of peers, teachers, and family members as sources of motivation. Thus, satisfaction of relatedness and belonging can enhance perceived social approval and normative pressure to engage in socially valued behaviors, providing theoretical support for the hypothesis that psychological need satisfaction positively predicts subjective norms.

### 2.4 The present study

Building upon this integrative framework, this study proposes a structural model that examines how satisfaction of five psychological needs–autonomy, competence, relatedness, novelty, and variety–predicts exercise intention among Chinese university students using campus running applications. In addition, this study explores whether this relationship is mediated by three TPB constructs: attitude, subjective norms, and perceived behavioral control.

By integrating both motivational and cognitive predictors, the model offers a more comprehensive explanation of technology-assisted exercise behavior, particularly in digitally saturated learning environments, where both internal motivation and belief-based reasoning are essential for sustained engagement.

**Figure 1:**
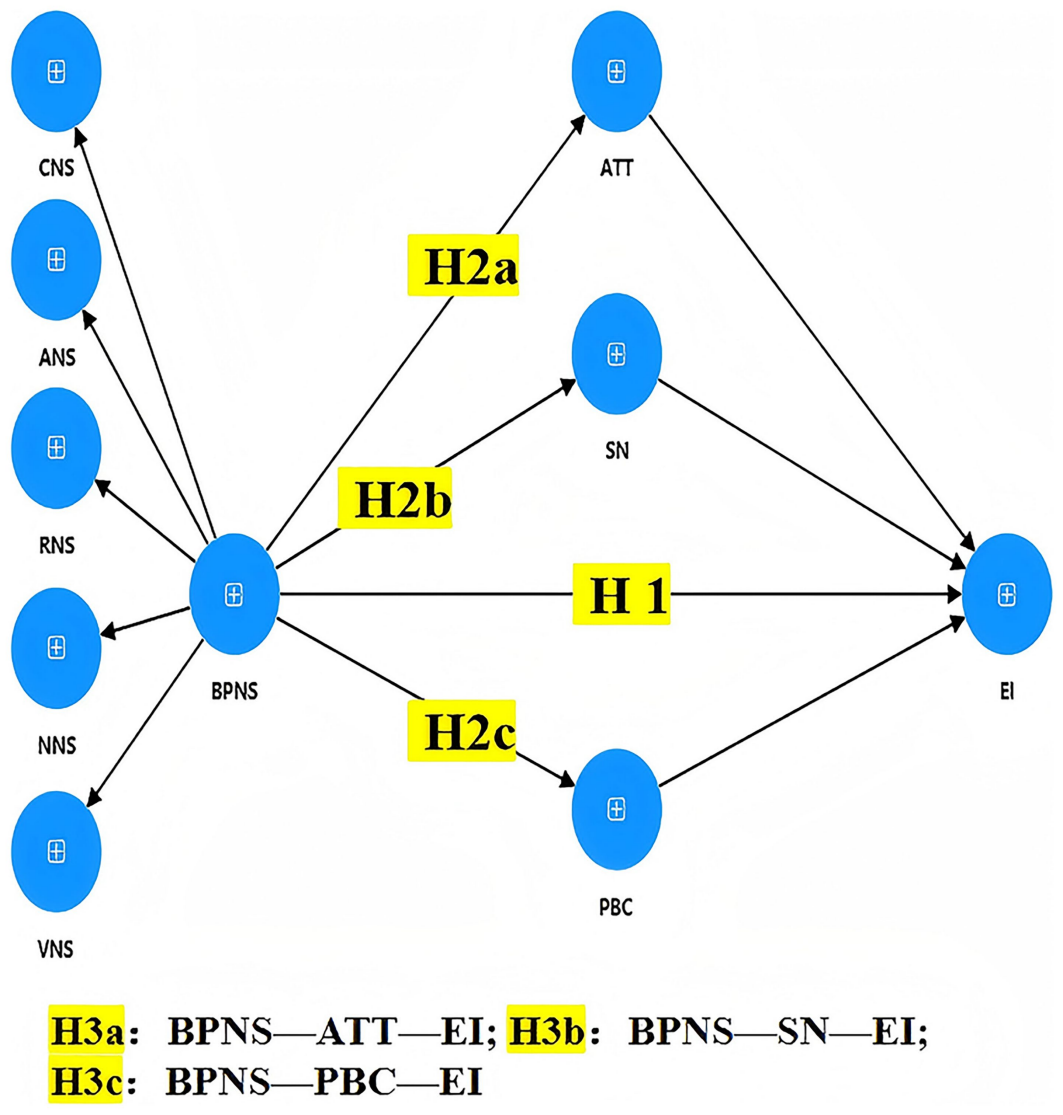
Path model of the hypothesized relationships.

Building on the integrated SDT–TPB framework, this study proposes the conceptual model shown in Figure 1. All study variables, their abbreviations, theoretical origins, and operational definitions are summarized in Table 2. Guided by this model, the following hypotheses are proposed:

**Table 2:** Study variables: abbreviations, theoretical sources, and operational definitions.

Abbr.	Full term	Theory	Operational definition
ANS	Autonomy-need satisfaction	SDT	The degree to which a student feels that using the campus running application is self-chosen and personally endorsed.
CNS	Competence-need satisfaction	SDT	The extent to which the student feels effective and capable when completing app-assigned running tasks.
RNS	Relatedness-need satisfaction	SDT	The degree of feeling cared for, accepted, and emotionally connected with others through the app’s social features.
NNS	Novelty-need satisfaction	Extended SDT	The extent to which the application offers new and stimulating experiences—unfamiliar routes, challenges, or functions.
VNS	Variety-need satisfaction	Extended SDT	The perceived diversity of activities and content within the application prevents monotony.
BPNS	Basic psychological-need satisfaction	SDT	A latent construct reflects overall fulfillment of ANS, CNS, RNS, NNS, and VNS in the digital exercise setting.
ATT	Attitude toward technology-assisted exercise	TPB	The student’s overall positive or negative evaluation of using the campus running app.
SN	Subjective norms	TPB	Perceived social pressure from important others (e.g., peers, family, coaches) to use the application for exercise.
PBC	Perceived behavioral control	TPB	The perceived capability to overcome time, physical, and environmental barriers and run with the app.
EI	Exercise intention	TPB	The intention strength, consistency, and frequency of anticipated future use.

*H1*: Basic psychological needs satisfaction (autonomy, competence, relatedness, novelty, and variety) positively predicts exercise intention.

*H2a*: Basic psychological needs satisfaction positively predicts attitudes toward technology-assisted exercises.

*H2b*: Basic psychological needs satisfaction positively predicts subjective norms.

*H2c*: Basic psychological needs satisfaction positively predicts perceived behavioral control.

*H3a*: Attitude mediates the relationship between basic psychological needs satisfaction and exercise intention.

*H3b*: Subjective norms mediate the relationship between basic psychological needs satisfaction and exercise intention.

*H3c*: Perceived behavioral control mediates the relationship between basic psychological needs satisfaction and exercise intention.

## 3 Method

### 3.1 Participants and procedure

This study adopted a purposive sampling strategy targeting full-time undergraduate students who had prior experience using campus running check-in applications, a form of technology-assisted exercise widely implemented in Chinese universities. A total of 500 questionnaires were distributed across 5 comprehensive universities in Shanxi Province, China, through recruitment volunteers on the universities’ intranets and bulletin boards, and filled out online questionnaire links using the Wenjuanxing platform. Recruitment information included survey invitations, consent forms, researchers’ contact details, and a QR code link to an electronic questionnaire. To ensure the uniformity of the sample distribution, 25 students were recruited for each grade at each university. Of the total distributed questionnaires, 491 were returned, and after screening for incomplete responses and invalid cases (*n* = 14), 477 valid responses were retained for data analysis, resulting in a valid response rate of 95.4 percent.

The five universities were chosen based on three criteria:

1. Institutional participation in the national digital physical education initiative aligned with the “Healthy China 2030” strategy;
2. Diversity of academic disciplines, including humanities, sciences, and engineering; and
3. Established usage of campus running systems for student fitness assessment.

Shanxi Province was selected as the study site because of its early adoption of digital exercise monitoring programs, provincial-level support for smart sports innovation in higher education, and representativeness of northern China’s university demographic and educational context. The region also reflects a growing emphasis on integrating technology into health promotion, making it an appropriate setting for investigating technology-assisted exercise behaviors among university students.

The participants had diverse academic backgrounds and ranged in age from 18 to 24 years, corresponding to the age group most commonly associated with declining physical activity (see Table 3). Prior to participation, all students were informed about the research objectives and procedures, and written informed consent was obtained. Participation was voluntary and anonymous, and the respondents could withdraw at any time without penalties. No monetary incentives were provided. The study protocol was reviewed and approved by the University Research Ethics Committee (Approval No. UM. TNC2/UMREC_3656). All procedures complied with the ethical standards for human research.

**Table 3:** Sample demographics (*N* = 477).

Variable	Category	Frequency	Percentage (%)
Gender	Male	154	32.3
	Female	323	67.7
Academic year	Year 1	121	25.4
	Year 2	124	26.0
	Year 3	120	25.1
	Year 4	112	23.5
Academic discipline	Humanities and social sciences	162	34.0
	Science and engineering	189	39.6
	Business and management	81	17.0
	Arts and physical education	45	9.4

### 3.2 Instruments

The survey instrument was developed based on previously validated scales adapted to the context of technology-assisted physical activity. A total of 42 items were used to measure eight latent constructs, each using a 5-point Likert scale ranging from 1 (Strongly Disagree) to 5 (Strongly Agree). The following measurement scales were used:

Psychological Need Satisfaction in Exercise Scale (Wilson et al., 2006): This scale included three constructs from SDT, namely Competence Need Satisfaction (CNS, six items), Autonomy Need Satisfaction (ANS, six items), and Relatedness Need Satisfaction (RNS, six items).

Novelty Need Satisfaction Scale (González-Cutre et al., 2016): This scale assessed Novelty Need Satisfaction (NNS, six items) under the extended SDT framework.

Variety Need Satisfaction Scale (Sylvester et al., 2014): This scale measured Variety Need Satisfaction (VNS, five items), also part of the extended SDT.

Theory of Planned Behavior Questionnaire on Physical Activity (Tirado-González et al., 2012): this questionnaire measured four constructs: Attitude (ATT, six items), Subjective Norms (SN, four items), Perceived Behavioral Control (PBC, five items), and Exercise Intention (EI, four items).

All items were carefully adapted to the digital exercise context and translated through a back-translation process to ensure conceptual equivalence. Two bilingual researchers independently translated the items into Chinese, an independent translator back-translated them into English, and discrepancies were reconciled in a consensus meeting with an expert panel in physical education and measurement. The questionnaire was then pilot-tested with 30 undergraduates from one university who had prior experience using the campus running check-in app. This pilot study followed three steps.

First, the participants completed the draft survey individually in quiet classroom conditions and marked any confusing words or examples. Second, cognitive debrief interviews were conducted in small groups of five to six students using think-aloud and probing questions that targeted comprehension, recall, judgment, and response mapping for each construct. Third, a short focus-group discussion gathered verbal feedback on clarity, relevance, and item length, and participants completed a one-page form rating clarity and relevance on a five-point scale and suggesting alternative wording. The research team summarized comments on the same day and revised the items according to predefined rules that prioritized conceptual fidelity, plain language, and consistency of response anchors.

Typical changes included replacing platform jargon with neutral terms; aligning Likert anchors across scales; clarifying referents in subjective norm items to peers, instructors, and physical education teachers; and simplifying perceived behavioral control statements to separate time, resource, and environmental barriers. No items were added or deleted at this stage. Minor linguistic and contextual revisions were finalized before the full survey administration. All pilot participants provided written informed consent, and the pilot study followed the same ethical approval as the main study.

### 3.3 Data analysis

Data screening and analysis were conducted using SPSS 26 and SmartPLS 4. Cases with more than 5 % missing values were removed, and multivariate outliers were identified using the Mahalanobis *D*^2^ statistic. The data distribution was checked before structural modeling, confirming that all variables conformed to normality.

Partial Least Squares Structural Equation Modeling (PLS-SEM) was employed following the recommendations of Hair et al. (2019). PLS-SEM was selected instead of covariance-based SEM because the present study focuses on theory development rather than theory confirmation and involves a moderately complex model containing both formative and reflective constructs. The PLS approach is also suitable for maximizing the explained variance in predictive models, which aligns with the exploratory nature of this research.

The analysis followed a two-stage process: first, the measurement model was evaluated, and second, the structural model was assessed. Reliability was examined using Cronbach’s alpha and composite reliability (CR), with CR calculated based on the rhoC (Dijkstra–Henseler’s reliability coefficient), which provides a more precise estimate of construct reliability in PLS models than Cronbach’s alpha. Convergent validity was verified through average variance extracted (AVE), and discriminant validity was assessed using both the heterotrait–monotrait ratio (HTMT) and the Fornell–Larcker criterion.

The structural model was tested using bootstrapping with 5,000 resamples to estimate the path coefficients, *t*-values, *p*-values, and effect sizes (*f*^2^). The predictive power (*R*^2^) and predictive relevance (*Q*^2^) were also reported to evaluate the explanatory and predictive strength of the model.

## 4 Results

### 4.1 Measurement model

Prior to testing the hypothesized structural relationships, the measurement model was evaluated to ensure the reliability and validity of all latent constructs. Following Hair et al.’s (2019) two-step PLS-SEM approach, the model was assessed for internal consistency, reliability, convergent validity, and discriminant validity.

Internal consistency reliability was evaluated using both Cronbach’s alpha (*α*) and composite reliability (CR). As shown in Table 4, all constructions exceeded the acceptable threshold of 0.70 for both indices, with all values surpassing 0.80. This indicates that the measurement items exhibited strong internal consistency and were suitable for representing their respective latent constructions. The constructions assessed included the five psychological needs—autonomy, competence, relatedness, novelty, and variety—the three TPB predictors (attitude, subjective norms, and perceived behavioral control), and exercise intentions.

**Table 4:** Cronbach’s alpha, CR, and AVE.

Constructs	Cronbach’s alpha (*α*)	Composite reliability (CR)	Average variance extracted (AVE)
ANS	0.895	0.919	0.656
ATT	0.888	0.916	0.648
CNS	0.923	0.940	0.724
NNS	0.901	0.925	0.673
EI	0.942	0.959	0.852
PBC	0.906	0.930	0.728
RNS	0.898	0.922	0.666
SN	0.934	0.953	0.836
VNS	0.914	0.936	0.745

Convergent validity was assessed through Average Variance Extracted (AVE). All constructions yielded AVE values greater than the recommended cutoff of 0.50, indicating that the majority of the variance in the observed indicators was accounted for by their respective latent variables. The measurement model demonstrated robust reliability and validity, ensuring that the indicators effectively represented their respective constructs.

Discriminant validity was evaluated using both the Fornell–Larcker criterion and the Heterotrait–Monotrait (HTMT) ratio. According to the Fornell–Larcker criterion, the square root of each construct’s AVE exceeded its correlation with the other constructs, supporting discriminant validity. Table 5 displays the Heterotrait-Monotrait Ratio (HTMT) values, which are used to assess discriminant validity among the constructs in the measurement model. HTMT values measure the correlation between different constructs, with values below 0.85 (or in some cases 0.90) indicating acceptable discriminant validity (Henseler et al., 2015). Most of the HTMT values in the table fall well below the threshold, confirming that the constructs are distinct.

**Table 5:** HTMT.

Constructs	ANS	ATT	BPNS	CNS	NNS	EI	PBC	RNS	SN	VNS
ANS										
ATT	0.231									
BPNS	0.840	0.304								
CNS	0.619	0.268	0.792							
NNS	0.547	0.251	0.913	0.541						
EI	0.376	0.718	0.435	0.371	0.344					
PBC	0.279	0.781	0.317	0.266	0.251	0.895				
RNS	0.572	0.145	0.857	0.460	0.720	0.231	0.142			
SN	0.239	0.804	0.321	0.231	0.286	0.718	0.833	0.215		
VNS	0.523	0.341	0.835	0.443	0.773	0.441	0.351	0.590	0.332	

A few constructs show relatively higher correlations (Table 6), such as EI and PBC (0.895) and BPNS and RNS (0.857), but these remain within acceptable limits, reflecting conceptual connections without compromising their distinctiveness (Henseler et al., 2015). In particular, the HTMT value between the BPNS and NNS is 0.913, which can be retained in order not to affect model validity (Franke and Sarstedt, 2019). In summary, the HTMT analysis confirms adequate discriminant validity across all constructions. Despite some moderate correlations, all values remain within acceptable thresholds, validating the measurement model’s robustness and ensuring that each construction is conceptually distinct.

**Table 6:** Discriminant validity based on the Fornell–Larcker criterion.

Constructs	ANS	ATT	BPNS	CNS	NNS	EI	PBC	RNS	SN	VNS
ANS	0.810									
ATT	0.209	0.805								
BPNS	0.770	0.282	0.656							
CNS	0.567	0.243	0.737	0.851						
NNS	0.495	0.230	0.856	0.500	0.821					
EI	0.345	0.667	0.419	0.351	0.322	0.923				
PBC	0.252	0.715	0.301	0.250	0.232	0.829	0.853			
RNS	0.520	0.111	0.792	0.427	0.652	0.222	0.128	0.816		
SN	0.218	0.744	0.309	0.218	0.269	0.674	0.769	0.202	0.914	
VNS	0.476	0.310	0.792	0.409	0.705	0.409	0.320	0.539	0.307	0.863

Together, these results confirm that the measurement model possesses satisfactory psychometric properties, including internal consistency, convergent validity, and discriminant validity. This validation provides a solid foundation for evaluating the proposed structural model, ensuring that the observed relationships among constructs reflect true theoretical associations rather than measurement errors.

### 4.2 Structural model

Following the confirmation of the measurement model’s reliability and validity, the structural model was evaluated to test the hypothesized relationships among the latent constructs. The analysis followed the PLS-SEM procedure recommended by Hair et al. (2019), using bootstrapping (5,000 resamples) to estimate the path coefficients, *t*-values, *p*-values, and coefficient of determination (*R*^2^).

Table 7 provides a detailed summary of the path coefficients (*β*), standard deviations (SD), *t*-values, *p*-values, and confidence intervals (CIs) for the integrated structural model, offering a comprehensive evaluation of the hypothesized relationships. Each path is assessed for statistical significance based on its *p*-value and whether the corresponding confidence interval excludes zero. The first hypothesis (H1) tests the direct effect of basic psychological need satisfaction (BPNS) on exercise intentions (EI). Similarly, Hypothesis 2 shows a significant positive relationship between basic psychological need satisfaction and attitudes (2a), subjective norms (2b), and perceived behavioral control (2c). Regarding the hypothesis, three indirect effects of attitudes (3a) and perceived behavioral control (3c) confirm a significant and substantial mediating effect. However, subjective norms (2b) are not statistically significant.

**Table 7:** Path coefficient, *t*-value, and *p*-values of the integrated model.

Hypothesis	Path	Path coefficient (*β*)	Standard deviations (SD)	*t* value	*p* value	Confidence interval		Significance
						2.50%	97.50%	
H1	BPNS → EI	0.177	0.032	5.550	0.000	0.113	0.239	Yes**
H2a	BPNS → ATT	0.282	0.055	5.144	0.000	0.179	0.392	Yes**
H2b	BPNS → SN	0.309	0.055	5.572	0.000	0.205	0.418	Yes**
H2c	BPNS → PBC	0.301	0.053	5.655	0.000	0.202	0.409	Yes**
H3a	BPNS → ATT → EI	0.037	0.016	2.359	0.018	0.012	0.071	Yes*
H3b	BPNS → SN → EI	−0.002	0.019	0.080	0.936	−0.040	0.035	No
H3c	BPNS → PBC → EI	0.207	0.036	5.670	0.000	0.138	0.280	Yes**

*p < 0.05; **p < 0.01.

#### 4.2.1 Model fit and explanatory power

Table 8 presents the *R*^2^ and Adjusted *R*^2^ values for the constructs in the integrated model, indicating the proportion of variance explained by the predictors for each dependent variable. These values reflect the explanatory power of the structural model and provide insights into the strength of the relationships among the constructions.

**Table 8:** Results of *R*^2^ of the integrated model.

Constructs	*R* square	*R* square adjusted
ANS	0.592	0.592
ATT	0.079	0.078
CNS	0.543	0.542
NNS	0.733	0.733
EI	0.726	0.724
PBC	0.091	0.089
RNS	0.626	0.626
SN	0.095	0.093
VNS	0.628	0.627

The construct exercise intention (EI) exhibits a high *R*^2^ value of 0.726, with an Adjusted *R*^2^ value of 0.724, indicating that approximately 72.4% of the variance in exercise intentions (EI) is explained by the predictors attitude (ATT), subjective norms (SN), and perceived behavioral control (PBC). This highlights the robustness of the model in predicting exercise intentions, with significant contributions from these key predictors.

Among the subcomponents of basic psychological need satisfaction (BPNS), novelty need satisfaction (NNS) achieves the highest *R*^2^ value of 0.733 and an Adjusted *R*^2^ value of 0.733, suggesting that basic psychological need satisfaction (BPNS) explains approximately 73.3% of the variance in novelty need satisfaction (NNS). Similarly, relatedness needs satisfaction (RNS) and variety needs satisfaction (VNS) display substantial *R*^2^ values of 0.626 and 0.626, respectively, indicating that basic psychological need satisfaction (BPNS) accounts for a significant proportion of the variance in relatedness and variety need satisfaction.

Competence needs satisfaction (CNS) and autonomy needs satisfaction (ANS) also demonstrate strong *R*^2^ values of 0.543 and 0.592, with Adjusted *R*^2^ values of 0.542 and 0.592, respectively, further confirming the dominant influence of basic psychological need satisfaction (BPNS) on competence and autonomy need satisfaction.

In contrast, the constructs attitude (ATT), subjective norms (SN), and perceived behavioral control (PBC) have relatively low *R*^2^ values. For instance, attitude (ATT) has an *R*^2^ of 0.079 (Adjusted *R*^2^ value = 0.078), while subjective norms (SN) and perceived behavioral control (PBC) show *R*^2^ values of 0.095 and 0.091, respectively, with slightly lower Adjusted *R*^2^ values. These results suggest that while basic psychological need satisfaction (BPNS) has a direct influence on these constructions, other unmeasured factors may also contribute to their variance.

The *R*^2^ values indicate that the integrated model is highly effective in explaining the variance in exercise intentions (EI) and the subcomponents of basic psychological need satisfaction (BPNS) (ANS, CNS, NNS, RNS, and VNS). The strong explanatory power of these constructions underscores the centrality of basic psychological need satisfaction (BPNS) in the model. However, the relatively low *R*^2^ values for attitude (ATT), subjective norms (SN), and perceived behavioral control (PBC) suggest that additional factors beyond basic psychological need satisfaction (BPNS) might be influencing these predictors. Overall, the results validated the structural model’s effectiveness in capturing the dynamics of basic psychological needs satisfaction and exercise intentions.

Table 9 presents the *Q*^2^ values for the dependent constructs in the integrated model, computed using the Stone–Geisser test with the formula *Q*^2^ = 1 – (SSE/SSO), where SSE denotes the sum of squared prediction errors and SSO represents the sum of squares of observations. *Q*^2^ values assess the out-of-sample predictive relevance of the model. A value above zero indicates that the model has predictive relevance for a given construct, whereas values closer to one denote stronger predictive accuracy (Hair et al., 2021).

**Table 9:** Results of *Q*^2^ of the integrated model.

Constructs	*Q*^2^ (=1−SSE/SSO)
ANS	0.384
ATT	0.050
BPNS	0.000
CNS	0.388
NNS	0.487
EI	0.613
PBC	0.065
RNS	0.411
SN	0.078
VNS	0.462

The results reveal meaningful variation in predictive relevance across constructs. The exercise intention (EI) construct exhibited the highest *Q*^2^ value (0.613), suggesting that the predictors—attitude (ATT), subjective norms (SN), and perceived behavioral control (PBC)—collectively offer strong predictive power for explaining students’ continued engagement with the campus running check-in app.

The subcomponents of the BPNS also demonstrated substantial predictive relevance. These findings indicate that the integrated model effectively captures the motivational mechanisms underlying the five dimensions of need satisfaction, further validating the inclusion of novelty and variety as essential constructs in digital health contexts.

In contrast, the predictive relevance of the three TPB variables was relatively limited. While all values remain above zero—indicating the model does provide some predictive power for these constructs—the relatively low scores suggest that additional antecedents or contextual moderators may be necessary to more effectively explain these proximal cognitive variables. Factors such as past behavior, habit strength, or peer influence may help increase the model’s precision for these components.

Taken together, the *Q*^2^ results underscore the robust predictive utility of the integrated SDT–TPB model, particularly in accounting for exercise intention and the five subdimensions of BPNS. However, the modest predictive relevance of ATT, SN, and PBC points to potential theoretical limitations and highlights the need for model refinement through the inclusion of additional psychological or contextual variables. These findings confirm the central role of psychological need satisfaction in technology-assisted health behavior while simultaneously identifying areas for further development within the TPB framework.

To complement the interpretation of path significance and explanatory power, effect sizes (*f*^2^) were calculated to determine the relative impact of each exogenous construct on its respective endogenous target. Table 9 summarizes the *f*^2^ values for the key paths in the model. According to Cohen’s (1988) guidelines, values above 0.02, 0.15, and 0.35 indicate small, medium, and large effects, respectively.

For instance, perceived behavioral control (PBC) demonstrated a medium effect on exercise intention (*f*^2^ = 0.621), whereas attitude (*f*^2^ = 0.024) and subjective norms (*f*^2^ = 0.000) exhibited small effects. These findings reinforce the structural importance of PBC in driving exercise intentions. Similarly, the BPNS showed small to moderate effect sizes on all three TPB constructs (*f*^2^ = 0.086 to 0.105), supporting the proposed mediation framework.

Table 10 presents the *f*^2^ values that quantify the local effect sizes of the individual predictor variables on their corresponding dependent constructs in the integrated model. These values represent each predictor’s unique contribution to the explained variance (*R*^2^) and provide additional insight into the strength and meaningfulness of structural relationships. Following Cohen’s (1988) guidelines, *f*^2^ values of 0.02, 0.15, and 0.35 represent small, medium, and large effects, respectively.

**Table 10:** Results of *f*^2^ of the integrated model.

Constructs	ANS	ATT	BPNS	CNS	NNS	EI	PBC	RNS	SN	VNS
ANS										
ATT						0.024				
BPNS	1.454	0.086		1.188	2.748	0.102	0.100	1.677	0.105	1.688
CNS										
NNS										
EI										
PBC						0.621				
RNS										
SN						0.000				
VNS										

The analysis reveals that basic psychological need satisfaction (BPNS) plays a central and dominant role across the model. Notably, BPNS exhibits an exceptionally large effect on novelty need satisfaction (NNS), with an *f*^2^ value of 2.748, indicating that BPNS overwhelmingly determines students’ perceived novelty experiences when using campus running check-in applications. Similarly, large effect sizes are observed for variety need satisfaction (VNS, *f*^2^ = 1.688) and relatedness need satisfaction (RNS, *f*^2^ = 1.677), affirming BPNS as a robust predictor of motivational freshness and social connectedness.

BPNS also exerts a strong influence on competence need satisfaction (CNS, *f*^2^ = 1.188) and autonomy need satisfaction (ANS, *f*^2^ = 1.454), underscoring its broad functional importance in fostering a range of internal motivational states. These findings collectively validate that BPNS functions as a higher-order latent construct, effectively accounting for the variability in all five subdimensions of psychological needs (ANS, CNS, NNS, RNS, and VNS).

In contrast, the effect of BPNS on proximal TPB variables is comparatively modest. For example, BPNS shows small effect sizes on attitude (ATT, *f*^2^ = 0.086) and perceived behavioral control (PBC, *f*^2^ = 0.100), suggesting that while psychological needs may shape cognitive beliefs, other factors beyond motivation—such as contextual cues or social influence—may also be at play. Likewise, the direct effect of BPNS on exercise intention (EI) is moderate (*f*^2^ = 0.102), implying that this relationship may be partially mediated by TPB variables, particularly PBC.

Among the TPB constructs, perceived behavioral control (PBC) emerges as the most influential predictor of exercise intention, with a very large effect size (*f*^2^ = 0.621). This underscores the importance of students’ perceived ease or difficulty in maintaining regular activities via digital tools. Attitude (ATT), on the other hand, demonstrates a small effect (*f*^2^ = 0.024), while subjective norms (SN) show no effect (*f*^2^ = 0.000), highlighting the negligible role of perceived social expectations in shaping exercise intention within this digital context.

Finally, although ANS, RNS, and VNS were not modeled as direct predictors in the structural paths, their high *R*^2^ values and large *f*^2^ values for BPNS reinforce the theoretical coherence and explanatory power of the motivational layer in the model.

Overall, the effect size analysis emphasizes the central importance of BPNS in supporting a wide range of motivational outcomes, particularly novelty and variety, which are especially salient in technology-assisted exercise contexts. Additionally, PBC stands out as the most decisive cognitive predictor of behavioral intention, validating the integration of TPB constructs. The lack of impact from SN suggests limited peer or normative pressure in students’ app-based physical activity choices, potentially due to the individualized nature of digital exercise. These findings not only support the theoretical model but also offer actionable insights for improving digital health interventions through the targeted design of motivational and control-supportive features.

## 5 Discussion

This study aimed to integrate SDT and TPB to examine how psychological need satisfaction influences exercise intention among university students using a campus running check-in application. The results validated the reliability and construct validity of the measurement model and confirmed the explanatory and predictive power of the structural model. The following discussion interprets the results from theoretical, cultural, and practical perspectives, addressing both significant and non-significant findings.

The findings confirmed that the integrated SDT–TPB framework effectively explains students’ technology-assisted exercise intention. Basic psychological need satisfaction (BPNS) had a significant positive influence on exercise intention (H1), consistent with SDT’s premise that fulfilling autonomy, competence, and relatedness fosters internal motivation (Deci and Ryan, 2000). The inclusion of novelty and variety as extended needs further strengthened the framework’s relevance in digital contexts, demonstrating that dynamic and diverse experiences are crucial for sustaining engagement in app-based exercise environments (González-Cutre et al., 2016).

Consistent with the proposed model, BPNS positively influenced attitude, perceived behavioral control (PBC), and subjective norms (SN). Among the three TPB variables, PBC emerged as the strongest mediator and the most powerful predictor of intention, aligning with prior studies emphasizing the central role of control beliefs in digital health behavior (Hagger et al., 2002). Attitude also contributed significantly, reflecting students’ positive evaluations of digital exercises as convenient, flexible, and beneficial.

However, subjective norms showed a non-significant effect on exercise intention. Within the TPB framework, SN is traditionally viewed as a key determinant of behavioral intention; however, this finding suggests a context-dependent shift. The nature of mobile health apps, characterized by personalization, anonymity, and individual goal setting, may reduce the visibility of social comparison and social pressure, thereby weakening the role of normative influence. In digital environments, users’ decisions are often self-regulated and autonomous rather than externally driven. This result also resonates with recent extensions, such as UTAUT2, which acknowledges that perceived social influence becomes less prominent when technology adoption reaches maturity or when usage behaviors are individualized (Venkatesh et al., 2012). Theoretically, this finding expands the TPB framework by indicating that subjective norms may play a diminished or situational role in digital exercise contexts, where autonomy and competence are more dominant motivational forces.

The cultural and institutional context of Shanxi Province provides meaningful insights into the interpretation of these findings. University students in northern China often experience academic intensity and strong institutional supervision in physical education. Many universities implement campus running check-in systems as part of their health assessment requirements. These systems, while promoting accountability, often incorporate reward-and-punishment mechanisms such as attendance-based scores or fitness points. Such external structures may strengthen perceived behavioral control by establishing clear expectations and routine engagement but may simultaneously weaken intrinsic motivation due to external enforcement.

Furthermore, teacher-student dynamics and campus exercise culture are likely to shape behavioral intentions. In traditional university settings, physical education instructors act as authority figures who encourage compliance rather than intrinsic enjoyment, which may explain the strong influence of control beliefs (PBC) and the weaker role of social norms. Additionally, the relatively uniform implementation of check-in systems across institutions may have reduced social differentiation, further diluting normative influence. These factors highlight the interaction between institutional structure and psychological mechanisms, emphasizing that contextualized digital exercise behaviors are influenced not only by personal motivation but also by educational and cultural environments.

While the structural model demonstrated satisfactory explanatory power, the analysis was conducted on the full sample without examining subgroup differences. Variables such as gender, academic year, and prior exercise experience could moderate the relationships between SDT and TPB constructs. For instance, previous studies have shown that female students may place greater emphasis on relatedness and peer support, whereas male students prioritize competence and control (Teixeira et al., 2012). Similarly, first-year students may respond differently to institutional regulations compared with senior students who have more autonomy.

Future research could apply multi-group analysis (MGA) or latent class segmentation to explore potential heterogeneity across subgroups. If longitudinal or larger samples are available, researchers may further investigate the temporal changes in motivation and the evolving effects of digital engagement. Although subgroup analysis was not feasible in the current dataset, acknowledging this limitation clarifies the boundaries of the model’s explanatory power and highlights promising directions for subsequent inquiry.

Beyond the integrated SDT–TPB framework, several external factors may influence students’ exercise intentions and warrant inclusion in future models. Gamification elements within apps, such as badges, leaderboards, and progress tracking, can reinforce users’ sense of competence and engagement (Hamari and Koivisto, 2015). Digital health literacy, prior exercise habits, and attitudes toward technology are also important predictors of behavioral intention. Incorporating these variables could extend the model’s contextual applicability and strengthen its predictive accuracy in diverse digital learning environments.

From a practical perspective, these findings suggest several strategies for educators and developers. First, to enhance perceived behavioral control, universities could provide flexible scheduling, improved infrastructure, and user guidance that reduce technical and environmental barriers. Second, to foster intrinsic motivation, application designers may integrate features that promote autonomy (personalized goal setting), competence (adaptive difficulty levels), and relatedness (peer interaction and support communities). Third, promoting novelty and variety through changing routes, seasonal challenges, and social campaigns can help maintain long-term user engagement. These strategies align with the Healthy China 2030 Vision by encouraging sustainable exercise habits and supporting the physical and psychological well-being of university students.

In summary, this study demonstrates that integrating SDT and TPB offers a robust theoretical framework for explaining technology-assisted exercise behavior among university students. While BPNS exerts a significant effect on both motivational and cognitive predictors, the diminished role of subjective norms highlights a contextual shift toward individual autonomy in digital health participation. The discussion further underscores the influence of cultural and institutional environments in shaping behavioral control and motivation. By identifying contextual, moderating, and external factors, this study not only contributes to theoretical advancement but also provides practical insights for the design of more engaging and motivationally supportive digital exercise systems.

## 6 Conclusion

This study investigated the psychological mechanisms underlying college students’ intentions to engage in technology-assisted exercise through an integrated framework combining Self-Determination Theory (SDT) and the Theory of Planned Behavior (TPB). By incorporating five psychological needs and three cognitive predictors, this study examined their direct and mediating effects on exercise intention within the context of a campus running check-in application. The findings confirmed that the satisfaction of basic psychological needs, particularly the extended needs for novelty and variety, plays a pivotal role in fostering exercise intention. Among the TPB variables, perceived behavioral control emerged as the most influential predictor, whereas subjective norms exhibited no significant effect.

The integration of SDT and TPB in this study contributes to the refinement of behavioral theory in digital health contexts. The results demonstrate that motivational satisfaction derived from SDT serves as a distal driver that shapes the cognitive determinants specified in TPB. This finding supports and extends previous integrative models by empirically validating the SDT–TPB pathway within a digital exercise environment. Moreover, the inclusion of novelty and variety expands the conceptual boundaries of SDT by recognizing that sustained engagement in repetitive digital activities depends on stimulation, diversity, and experiential renewal. These mechanisms clarify why traditional models focusing solely on autonomy, competence, and relatedness may not fully explain persistence in technology-mediated exercises. Theoretically, this study enriches the understanding of how intrinsic motivation interacts with belief-based cognition, offering a more holistic framework for predicting and promoting sustained digital health behaviors.

These findings provide insights into the psychological mechanisms underlying digital exercise behavior. Novelty and variety act as motivational catalysts that prevent psychological saturation and sustain user engagement through cognitive freshness and emotional stimulation. When students encounter new challenges, interactive routes, or varied activity modes, they experience a sense of renewal that reinforces their competence and autonomy. This dynamic aligns with the principles of experiential learning and behavioral reinforcement, suggesting that engagement in digital physical activity is not static but evolves with the quality and diversity of user experience. Accordingly, digital platform designers should embed adaptive features such as personalized feedback, evolving goals, and diverse activity options to satisfy these motivational needs and promote sustained participation.

Beyond application design, the findings carry implications for higher education policy, teaching practice, and health promotion. Universities should integrate digital exercise programs into physical education curricula, not merely as monitoring tools, but as platforms that foster self-determined motivation. Policies that reward intrinsic engagement rather than mere compliance can enhance long-term behavioral adherence. Educators and health administrators can employ motivational strategies that balance structure with autonomy, allowing students to internalize health-promoting behaviors. At the societal level, insights from this study may inform the design of broader digital health initiatives under the Healthy China 2030 strategy, emphasizing technology’s role in reducing inactivity among young adults and bridging disparities in health behavior across regions and populations.

Although this study offers both theoretical and practical contributions, certain limitations should be acknowledged. The sample was drawn from university students in Shanxi Province, which may limit the generalizability of the findings to other cultural or demographic contexts. The cross-sectional design constrains the ability to infer causality or observe motivational changes over time. Future studies should employ longitudinal or experimental approaches to examine how psychological need satisfaction evolves with continued digital engagement. Furthermore, future research could conduct cross-cultural or cross-platform validations to test the universality of the integrated SDT and TPB models. The relatively limited explanatory power of attitude and subjective norms also suggests that additional constructs, such as digital health literacy, emotional engagement, and habit strength, may enrich the theoretical model. Examining these factors would further clarify the dynamic interplay between motivation, cognition, and context in digital exercise participation.

In conclusion, this study advances the theoretical understanding and practical knowledge of technology-assisted exercise behavior by integrating motivational and cognitive perspectives. This demonstrates that fulfilling basic psychological needs, especially novelty and variety, together with enhancing perceived behavioral control, can effectively promote students’ engagement in digital physical activity. The integrated SDT and TPB framework proposed here contributes to both behavioral theory and applied health research by illustrating how motivational satisfaction drives intention formation in technology-rich environments. These insights provide valuable guidance for educators, policymakers, and designers seeking to develop more engaging, equitable, and sustainable digital exercise systems that support the well-being and lifelong health of young adults.

## Data Availability

The raw data supporting the conclusions of this article will be made available by the authors, without undue reservation.
